# Revealing active components, action targets and molecular mechanism of Gandi capsule for treating diabetic nephropathy based on network pharmacology strategy

**DOI:** 10.1186/s12906-020-03155-4

**Published:** 2020-11-23

**Authors:** Qiqiang Zhang, Qing Ye, Xiaohui Huang, Ajing Xu, Yan Liu, Jia Qi, Hai Zhang, Jian Zhang

**Affiliations:** 1 Department of Pharmacy, Xinhua Hospital, Affiliated to Shanghai Jiao Tong University, School of Medicine, Shanghai, 200092 China; 2 Department of Pharmacy, Shanghai First Maternity and Infant Hospital, Tongji University School of Medicine, Shanghai, 201204 China

**Keywords:** Gandi capsule, Network pharmacology; active component UHPLC-QQQ-MS/MS, Surface plasmon resonance, Molecular mechanism; action target

## Abstract

**Background:**

Gandi capsule is a traditional Chinese herbal formula used to promote blood circulation and removing blood stasis in clinical. Our previous study has shown that it reduces proteinuria with routine treatment in diabetic nephrophy (DN), but its pharmacological action mechanism is still unknown.

**Methods:**

To facilitate the identification of components, a component database of Gandi capsule and target database of DN were established by ourselves. The components absorbed in blood circle were identified in rat plasma after oral administration of Gandi capsule by UHPLC-QQQ-MS/MS. The potential targets were screened by using Libdock tolls in Discovery studio 3.0. Then Pathway and Network analyses were used to enrich the screened targets. The possible targets were verified by using a surface plasmon resonance (SPR) test and the molecular mechanism focusing these targets for treating DN was clarified by western blot.

**Results:**

Six components in Gandi capsule were identified detected in rat plasma after oral administration by UHPLC-QQQ-MS/MS. After molecular docking analyses in KEGG and Discovery studio, four protein targets including HNF4A, HMGCR, JAK3, and SIRT1, were screened out, and proved as effective binding with baicalin, wogonoside by SPR. And the molecular mechanism was clarified that baicalin and wogonoside inhibit the effect of high glucose (HG)-induced decreased cell viability and podocin expression, and strengthen the activation p-AKT, p-PI3K, and p-AMPK.

**Conclusion:**

Baicalin and wogonoside were screened out to be the active compounds in Gandi capsule and can ameliorate HG-induced podocyte damage by influencing the AMPK and PI3K-AKT signaling pathways by binding with HNF4A, HMGCR, JAK3, and SIRT1.

**Graphical abstract:**

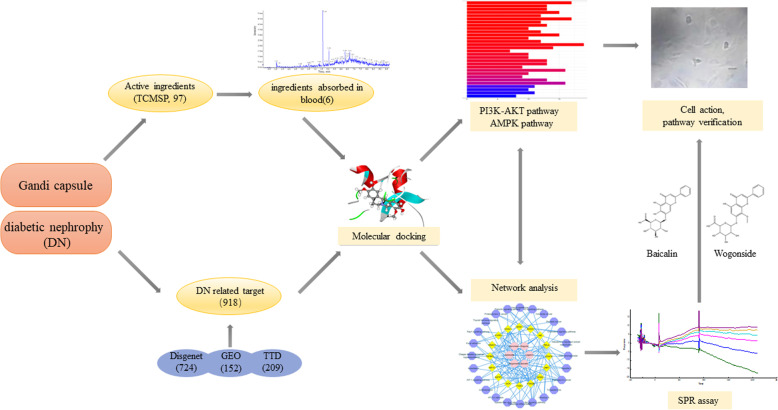

**Supplementary Information:**

The online version contains supplementary material available at 10.1186/s12906-020-03155-4.

## Background

Diabetic nephropathy (DN) is a common diabetic complication that can lead to end-stage renal disease [1], also causes glomerulonephritis [2]. The morbidity and mortality related to DKD has rapidly increased over the past 20 years [3]. According to previous epidemiological data, DKD could be the primary cause for the progression to dialytic end-stage kidney diseases in the urban population in China [4]. The common clinical features of DN are proteinuria, progressive renal impairment, hypertension, and edema [5]. In clinic, hypoglycemic agents, lipid-lowering drugs, and anti-vasoactive drugs are the conventional medicines for DN [6]. Some of these drugs, including the sodium-dependent glucose transporter 2 (SGLT-2) inhibitor and angiotensin-converting enzyme inhibitor (ACEI), have been shown to treat proteinuria [7–9].

Gandi capsule (GDC) is derived from traditional Chinese medicine (TCM) prescription and used to promote blood circulation and assist the regular therapy of DN [10]. The GDC is composed of eight varieties of traditional Chinese herbs: *Astragali Radix (Astragalus mongholicus Bunge., Huangqi)*, *Corni Fructus (Cornus officinalis Siebold & Zucc, Shanzhuyu)*, *Rehmanniae Radix (Rehmannia glutinosa Gaertn., Dihuang)*, *Leonuri Herba (Leonurus japonicus Houtt., Yimucao)*, *Sophorae Flos (Styphnolobium japonicum (L.) Schott., Huaihua)*, *Scutellariae Radix (Scutellaria baicalensis Georgi., Huangqin)*, *Bombyx Batryticatus (Bombyx scindicus (Stocks) I.Riedl., Jiangcan)*, and *phyllanthi fructus (Phyllanthus emblica L., Yuganzi)* and full information of GDC is shown in Table S1. In our previous study, GDC reduced proteinuria in combination with routine medicinal therapy during a 4 month observation period [10]. Through metabonomic analysis on blood and urine of diabetes patients treated by GDC for 6 months, it was found that the levels of urinary albumin excretion was decreased and the levels of estimated glomerular filtration rate (eGFR) was increased after treatment of GDC [11]. Moreover, we have analyzed the compounds in GDC by UHPLC-QQQ-MS/MS in our previous research [12]. Considering the complex of components in GDC, research on the pharmacological mechanism of GDC is still a challenge. There is a urgent need to clarify the active components, action targets and molecular mechanism.

Network pharmacology is based on systems biology and uses bioinformatic methods [13]. It integrates the advantage of systems biology at the level of the organism, is applicable to the analysis of complex mixtures, and has been widely used for the analysis of TCMs [14], especially in drug discovery [15], synergistic pharmacology [16], and compatibility mechanism analysis [17]. In our previous study, we used surface plasmon resonance (SPR) technology to discover the interactions of molecules and protein targets in vitro, which is a tool used to observe the interactions and dynamic changes between biological molecules. When a molecule binds to a protein on the surface of a medium, the refractive index of the medium will change, which can be transferred to an electrical signal. After the analysis of this signal, a series of parameters are displayed to evaluate the combination [18].

In this study, based on the clinical efficacy and component analysis of the Gandi capsule, a predicted analysis method was applied to screen active components and their potential targets via network pharmacology and structural biology, and further clarify the molecular mechanism. The results could provide a better understanding of the pharmacological mechanism of GDC in the treatment of DN.

## Methods

### Reagents and materials

Gandi capsules was supplied by Fangxin Pharmaceutical Co., Ltd. (Shanghai, China). The batch number of Gandi capsules used are GD20190304 and manufacturing date is March, 16th, 2019. The standard powders, morroniside, loganin, baicalin, wogonoside, wogonin, and sweroside, were purchased from Nature Standard Co., Ltd. (Shanghai, China). Primary antibodies against rabbit podocin, phosphoinositide 3-kinase (PI3K), p-PI3K, protein kinase B (AKT), p-AKT, 5′ AMP-activated protein kinase (AMPK) were from Abclone (Shanghai, China), and the dilutions were 1:1000. The primary antibodies against rabbit p-AMPK was from Abcam (Cambridge, USA) and dilution was 1:2000. The anti-rabbit DyLight™ 680 conjugated secondary antibodies from CST (Massachusetts, USA) and dilution was 1:1000. Hepatocyte nuclear factor 4-alpha (HNF4A), 3-hydroxy-3-methylglutaryl-coenzyme A reductase (HMGCR), tyrosine-protein kinase Janus kinase 3 (JAK3), and NAD-dependent protein deacetylase sirtuin-1 (SIRT1) were obtained from Proteintech Co., Ltd. (Chicago, America). Recombinant interferon gamma was from Sigma (Aldrich, USA). CCK-8 Kit was from YEASEN (Shanghai, China). Radioimmunoprecipitation assay lysis buffer and Bicinchoninic acid protein assay kit were from Beyotime (Shanghai, China). Nitrocellulose membrane was from Pall Co., Ltd. (New York, USA).

### Experimental animals

All experimental procedures complied with the Guidelines on the Care and Use of Laboratory Animals (National Institutes of Health Publication no.85–23, revised 1996) and were approved by the Institutional Animal Care and Use Committee of the Shanghai Jiaotong University, School of Medicine [Shanghai, China, NO. SYXK (Shanghai) 2018–0038], and authorized by Xinhua Hospital, affiliated to Shanghai Jiao Tong University, School of Medicine (Shanghai 200,092, China). Male sprague Dawley (SD) rats at 10 weeks old, weighing 180-220 g, were supplied by Shanghai SLAC Laboratory Animal Co.,Ltd. (Shanghai, China).

All animals were fed in a SPF environment in the Laboratory Animal Center of Xinhua Hospital affiliated to Shanghai Jiaotong University School of Medicine maintained at the temperature of 25 ± 2 °C and relative humidity of 45 ± 5% and provided with water and food freely.

### Establishment of component database and target protein database

According to the composition of the Gandi capsule, we built a compound database with 136 components using a Traditional Chinese Medicine and Active Ingredient Database (http://www.organchem.csdb.cn/). After analyzing the oral bioavailability (OB) and drug-likeness (DL) in the traditional Chinese medicine systems pharmacology online database (http://lsp.nwu.edu.cn/). Filter parameters were OB > 30% and DL > 0.18. Finally, 97 components were considered for this study.

We also founded a Target Protein Database to contain the protein targets related to DN. These targets are divided into two parts: confirmed targets and predicted targets. Confirmed targets are from the online databases the Therapeutic Target Database (TTD) (http://bidd.nus.edu.sg/group/cjttd/) and DisGeNET (http://www.disgenet.org/) by searching the keyword “diabetic nephropathy”. Predicted targets came from microarray data GSE30528, which includes 9 DN samples and 13 diabetes mellitus samples. This data is from the Gene Expression Omnibus (GEO) database (https://www.ncbi.nlm.nih.gov/geo/). To select more accurate targets, we set the filter parameter to *P* ≤ 0.05 and log fold change (logFC) (differential multiple) ≥ 2 or logFC ≤ − 2. Finally, we synthesized two different targets for diabetes mellitus from the target protein database.

### Analysis of components absorbed in blood by UHPLC-QQQ-MS/MS

Before experiment, rats were fasted 12 h before experiment and water was taken freely. And then the contents of the GDC were made into a suspensions with sterile water-5%Tween 80.Total 12 rats were administered with 0.27 g of drug suspensions per 1 kg of bodyweight or sterile water randomly by way of stomach, each group had six samples. This dose was identified based on the clinic dosage (0.3 g/per capsule* 3 capsules/per time* 3 times/ per day) of GDC in patients weighting 60 kg. And according to the result of pre-expreiment, 2 h later, after all rats receving CO_2_ gently and sacrificing, blood samples were collected by heart exposing and punctures. Then the addition of 500 μL of acetonitrile and 10 μL of internal standard per 90 μL, the mixture was vortexed for 30 s and centrifuged at 13,000 rpm for 4 min. Only the supernatant was used. The standard powders, morroniside, loganin, baicalin, wogonoside, wogonin, and sweroside, were purchased from Nature Standard Co., Ltd. (Shanghai, China). Every drug powder was high purity (≥ 98%). The standard samples were added to a blank sample and operated by the same process as the capsule treated rats. The concentration of each standard compound in the mixture was 500 ng/mL.

To accurately identify the compounds, we compared the samples with standards via UHPLC-QQQ-MS/MS, which consisted of a Waters UPLC system with an Applied Biosystem 5500 QTRAP® hybrid triple-quadrupole mass spectrometer (Applied Biosystems/MDS Sciex, Foster City, CA, USA). This system was equipped with a turbo ion spray source. Chromatographic separation was performed on a ZORBAX Eclipse Plus C18 (50 mm × 2.1 mm, 1.8 μm). The mobile phase consisted of (A) 0.1% formic acid-deionized water and (B) acetonitrile. The autosampler tray was maintained at 20 °C and the column at 50 °C. The following gradient elutions were used: 0.0–3.0 min, 30.0 → 74.0% B; 3.0–3.1 min, 74.0 → 95.0% B; 3.1–4.6 min, 95% B; 4.6–4.7 min, 95.0 → 30% B; and 4.7–7.0 min, 30% B.

Nitrogen was used in four different ways: the curtain (CUR) gas, nebulizer (GS1), heater (GS2), and the collision activation dissociation (CAD) gas. Firstly, all targeted analytes were tested in the multiple reaction monitoring (MRM) acquisition mode by MS/MS for simultaneous detection and two precursor-to-product ion transitions were monitored for the two compounds. Then, all source settings and instrument parameters for each MRM transition were optimized for the generation of as much protonated analyte molecules ([M + H]^+^) as possible for each targeted compound to efficiently produce its characteristic fragment/product ions. The ion spray needle voltage was set at 5500 V and the source temperature was 550 °C.

The following setting parameters were optimized; electrospray ionization source was operated with the CUR, GS1, and GS2 set at 40, 35, and 35 psi, respectively; independent precursor-product ion transitions specific to each analyte that depended on the instrumental parameters of the compound, such as the precursor ion, two product ions, declustering potential (DP), entrance potential (EP), collision energy (CE), and collision cell exit potentials (CXP); and the parameters were 50 V, 19 eV, 10 V, and 12 successively for the lactones. To detect osthol, the transition from precursor ion [M + H]^+^ at m/z 233.2 to product ion at m/z 187.1 was monitored. As the transition from the precursor was m/z 245.1 to the product at m/z 189.1, the dwell time was set at 550 ms.

### Molecular docking

To establish the correlation between drug molecules and targets, we used the docking software in Discovery Studio 3.0. Firstly, all target names were standardized using the data from the Uniprot database (https://www.uniprot.org/). Then, by selecting the minimum value from each resolution, a parameter used to describe the difference between the real and virtual target structures, the best target structures were obtained. All target structures were crystal structures determined by X-ray. This provided another filter for our study. A total of 373 protein targets and their structures were imported into Discovery Studio 3.0. After initial preparation, all structures were optimized by hydrogenation, water removal, or structural simplification. The canonical simplified molecular-input line-entry system data were imported into the software to build the molecular structures. Finally, every molecule was docked with the targets above to acquire the docking parameter X_libdockscore_. In addition, the ligand of each target was docked to acquire the parameter X_ligand_. All operations were performed under the CHARMM force field.

### Kyoto encyclopedia of genes and genomes (KEGG) enrichment analysis

To reduce the false-positive result of docking, two calculation formulas were applied. One was “D-value = X_libdockscore_ - X_ligand_ and D-value ≥ 0”, which was applied to confirm the differential binding affinity of molecules with targets compared to that with the ligand. The other was “^−^X_libdockscore_ = Σ(X_libdockscore_)/(number of targets) and X_libdockscore_ ≥^−^X_libdockscore_”. This formula filtered the term near the critical value. A calculation was performed for every molecule. After analyzing the docking data, all targets were subjected to KEGG enrichment analysis. This step was achieved using the online analysis website David (https://david.ncifcrf.gov/). We acquired enrichment pathways easily using the Function Annotation module. All enriched pathways were displayed in turn by comparing the *P* values.

### Network building and analysis

To establish the connection of molecules, targets, and pathways all selected terms were imported into Cytoscape 3.7 based on the results of molecular docking and KEGG enrichment analysis. Following the network analysis, an attached software analysis module was applied. Afterward, the parameter “Neighborhood Connectivity” (NC) was applied. As for the targets and pathways, if their NC was more than that of others, it was considered to be more enriched.

### Target validation by SPR assay

To verify the effectiveness of the above method, 11 representational molecule-target pairs were chosen for the SPR test. We installed 3 regulars to choose these 11 molecule-target pairs. The first one was ‘greater value of “Neighborhood Connectivity”’. The second one was ‘greater value of “X_libdockscore_”’. The last one was ‘target that docked with more molecules’. In the SPR test, the solution of protein standards was prepared and the concentration of the protein solution was 500 μg/mL. After combining with chips, a series of concentrations of compounds, which were double diluted step to step, were tested for their binding affinity to the proteins. Finally, the binding level between the protein and molecule was determined by the parameter Kd value.

### Cell line and cell culture

Human podocyte cell line was obtained from XYBiotechnology (Shanghai, China) and was cultured as previous research [19]. Briefly, cells were cultured in a Roswell Park Memorial Institute (RPMI) 1640 medium (Hyclone, Shanghai, China) mixed with 10% fetal bovine serum (FBS) and 1% penicillin-streptomycin at 5% CO_2_ and 37 °C. Before the experimental operation, the cells were cultured in RPMI 1640 medium supplemented with 10 U/mL recombinant interferon gamma at 33 °C for 2 weeks after seeding in culture plates. Only the differentiated cells were used in our research.

### Cell counting kit-8 (CCK-8) assay

Cells were shifted to a 96-well plate at a density of 1 × 10^5^/well. Afterwards, the cells were treated with a series of baicalin concentrations (0, 2.5, 5, 10, 12.5, 25, 50, 75, and 100 mg/L) and wogonoside (0, 2.5, 5, 10, 12.5, 25, 50, and 100 mg/L). After approximately 48 h, the samples were assayed with CCK-8 at 450 nm to find a maximum safe concentration for podocyte cells. Later, in another 96-well plate, cells were exposed to a high glucose condition (HG, 30 mM D-glucose), normal glucose condition (Control, 5 mM D-glucose), mannitol (MA, 5 mM D-glucose + 25 mM mannitol), dimethyl sulfoxide (DMSO, < 0.5%), baicalin (5 or 10 mg/L), and wogonoside (5 or 10 mg/L). This was also assayed with CCK-8 solution at 450 nm after 48 h.

### Western blot analyses

After incubation with the indicated conditions, the cells were washed twice with 1× phosphate-buffered saline and lysed with radioimmunoprecipitation assay lysis buffer. After vortexing 15 s and centrifugation in 14,000 g, the extracted protein content was detected using a bicinchoninic acid protein assay kit. Afterward, various groups of protein were electrophoresed using 10% sodium dodecyl sulfate-polyacrylamide gel electrophoresis and transferred onto a nitrocellulose membrane via electroblotting. To prevent non-specific binding, the membrane was blocked with 5% non-fat milk dissolved in 1× Tris-buffered saline and 0.1% Tween 20. Then, the membranes were incubated with primary antibodies against rabbit podocin, PI3K, p-PI3K, AKT, p-AKT, AMPK, and p-AMPK at 4 °C overnight. The membrane was then washed with 1 × TBST three times and incubated with DyLight™ 680 conjugated secondary antibodies (CST, Massachusetts, USA) for 1 h. Finally, after washing three times with 1 × TBST, the membrane was scanned.

### Statistical analysis

All experiments were repeated for three times. Statistical analysis was conducted using GraphPad Prism 7.0 software (La Jolla, CA, USA). The Student’s t test was used to examine the differences between two groups. Differences were considered significant if *P* < 0.05.

## Results

### Construction of molecule database and protein database

**Fig. 1:**
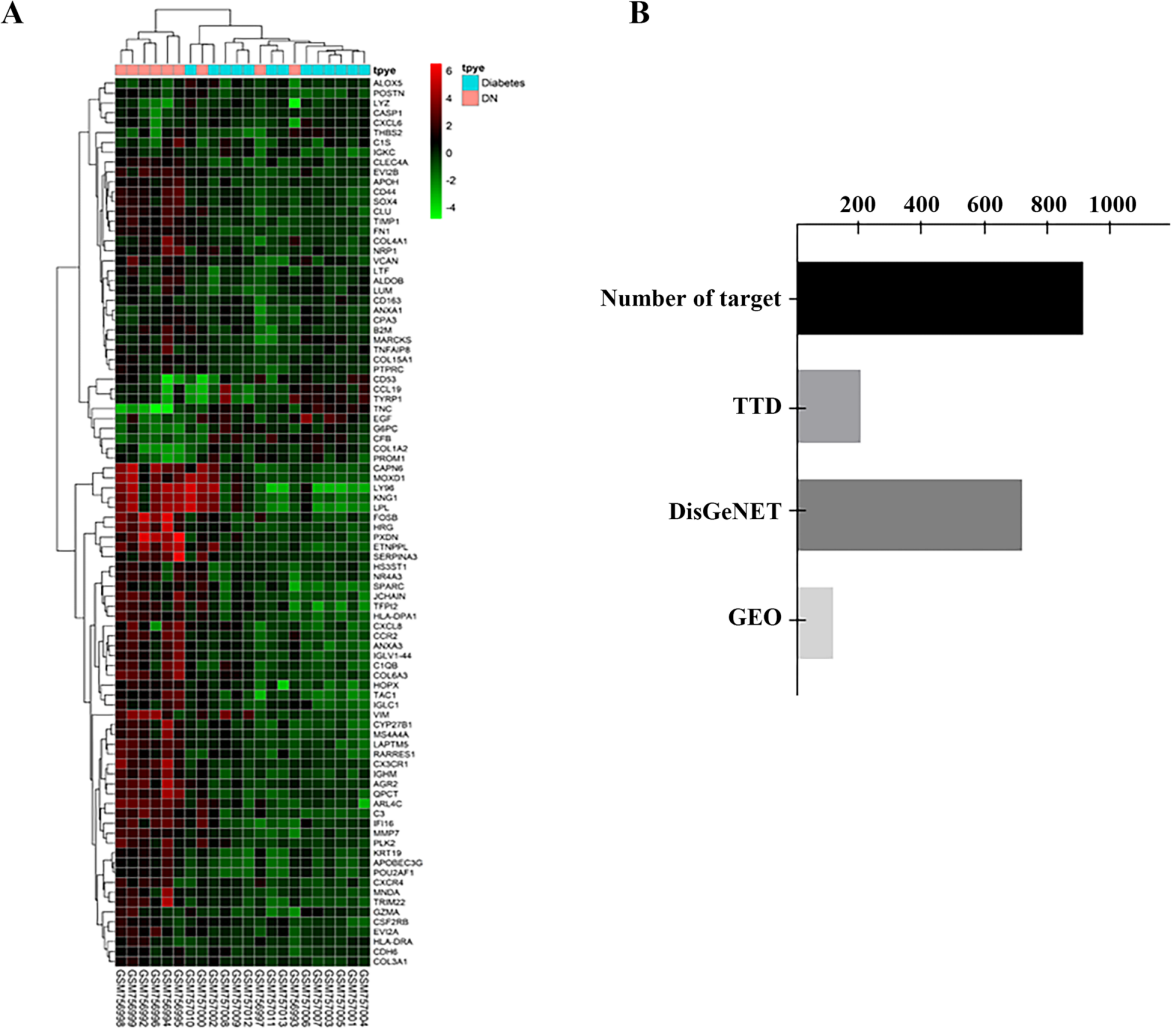
The molecular and protein databases. **a** Heatmap of microarray data “GSE30528”. The filter parameter is “*p* ≤ 0.05 and logFC ≥ 2 or logFC ≤ -2”. **b** The source constitution of the protein target database. TTD targets are from the online database “http://bidd.nus.edu.sg/group/cjttd/” and DisGeNET is from “http://www.disgenet.org/”. Moreover, the target using the term “GEObase” is from microarray data GSE30528

There were 97 components in the filtered molecule database (shown in Table S2). After comparison with 52 ingredients in Gandi capsules in vitro which we detected before [12], only 12 potential components were used, as shown in Table 1. As for predicted targets, there were 119 genes that changed after filtration, which means that these protein targets might be related to DN. The differential expression of these genes is shown in Fig. 1a. Additionally, there were 209 confirmed targets in TTD and 724 in Disgenet. After removing duplicates, there were 918 targets in the target protein database and the source constitution of the target protein database is shown in Fig. 1b.

**Table 1:** Information of 12 potential components

NO.	Molecule Name	Formula	MW	Mol ID	Origin
1	formononetin	C_16_H_12_O_4_	268.28	MOL000392	*Astragali Radix*
2	isorhamnetin	C_16_H_12_O_7_	316.28	MOL000354	*Astragali Radix*
3	kaempferol	C_15_H_10_O_6_	286.25	MOL000422	*Astragali Radix*
4	quercetin	C_15_H_10_O_7_	302.25	MOL000098	*Astragali Radix*
5	ellagic acid	C_14_H_6_O_8_	302.20	MOL001002	*Phyllanthi fructus*
6	digallate	C_14_H_9_O_9_	321.21	MOL000569	*Phyllanthi fructus*
7	loganin	C_17_H_26_O_10_	390.43	MOL001680	*Corni Fructus*
8	morroniside	C_17_H_26_O_11_	406.38	MOL001683	*Corni Fructus*
9	sweroside	C_16_H_22_O_9_	358.38	MOL000650	*Corni Fructus*
10	baicalin	C_21_H_18_O_11_	446.39	MOL002776	*Astragali Radix*
11	wogonoside	C_22_H_20_O_11_	460.40	MOL013068	*Scutellariae Radix*
12	wogonin	C_16_H_12_O_5_	284.28	MOL000173	*Scutellariae Radix*

### Identification of components absorbed into blood circle via UHPLC-QQQ-MS/MS

Both positive and negative ion detection modes responded well in quadrupole full scans by a 100 ng/mL tuning solution in methanol, which was achieved in positive ionization mode for isoalantolactone, alantolacton. The protonated molecular ions revealed peaks of morroniside, loganin, baicalin, wogonoside, wogonin, sweroside and internal standard (IS) at m/z 451.0, 408.2, 445.1, 461.1, 285.1, 357.1 and 245 respectively. Most abundant ions were produced by the target protonated molecular ions, and morroniside was observed in [M-HCOO]^−^ ions at m/z 179.0; loganin was in [M + NH_4_]^+^ at m/z 229.0; baicalin was in [M + H]^−^ at m/z 269.0; wogonoside and wogonin was in [M + H]^+^ at m/z 283.0 and 270.0 respectively; sweroside was in [M-H]^−^ at m/z 125.0. The mobile phase, types of chromatography columns, and column temperatures were tested to optimize the chromatographic conditions.

An acetonitrile-water mobile phase system from 30 to 95% acetonitrile without any acid in 3.10 min was applied because it can enhance the mass spectrum response of isoalantolactone and alantolactone. Zorbax Eclipse Plus-C18 (2.1 × 50 mm, 1.8 μm) analytical column was selected to produce more satisfactory chromatography results than other commercially available C18 columns, such as Poroshell 120EC-C18 (3.0 × 50 mm, 2.7 μm) and Acquity UPLC BEH C18 (2.1 × 50 mm, 1.7 μm). Three column temperatures (30 °C, 40 °C, and 50 °C) were tested and 50 °C was selected as it had the shortest retention time. Based on the above analysis, morroniside, loganin, baicalin, wogonoside, wogonin, sweroside were eluted at retention times of 3.106, 3.407, 4.592, 5.100, 6.300, and 3.490 min, respectively, and IS was eluted at 2.60 min. The parameters of target ions were set at m/z 451.0 → 179.0 for morroniside, m/z 408.2 → 229.0 for loganin, m/z 445.1 → 269.0 for baicalin, m/z 461.1 → 283.0 for wogonoside, m/z 285.1 → 270.0 for wogonin, m/z 357.1 → 125.0 for sweroside and m/z 245.1 → 189.1 for IS in the MRM mode for UHPLC-QQQ-MS/MS.

**Fig. 2:**
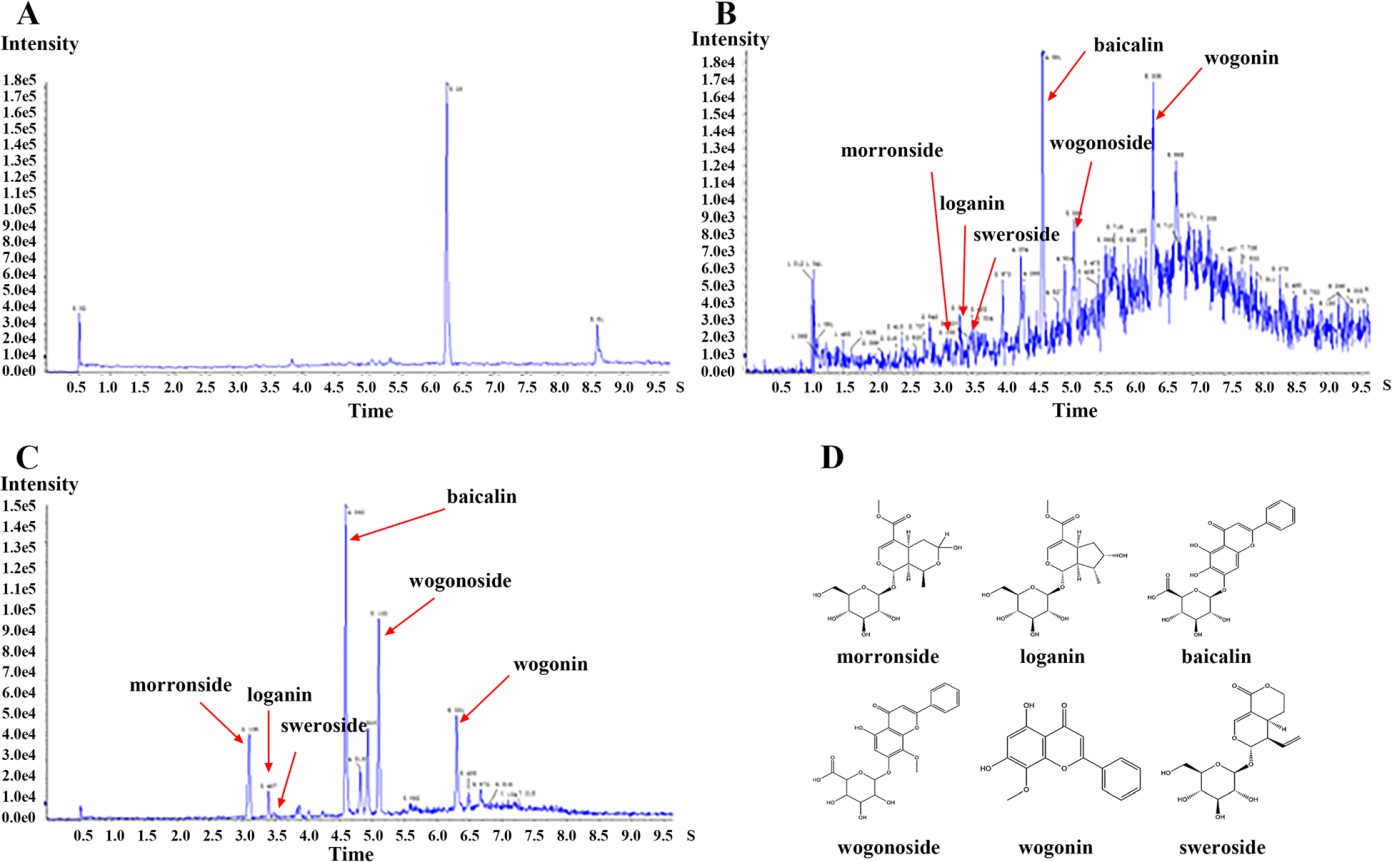
Ion flow diagram of UHPLC-QQQ-MS/MS. **a** Blank plasma **b** Plasma sample from rat after oral administration with Gandi capsule (12 g/kg) for 2 h **c** Standard mixture **d** Six potential compounds’ structures

After the analysis of the sample, six representational molecules, morroniside, loganin, baicalin, wogonoside, wogonin, and sweroside, were selected. The total ion chromatograms for the compounds are shown in Fig. 2a–d and their information is in Table 2.

**Table 2:** Mass spectrometry of six representational compounds

ID	Compounds	Formula	Molecular Weight (g·mol^−1^)	Retention Time t/min	Parent ion (m/z)	Product ion (m/z)	Ionization mode
1	morroniside	C_17_H_26_O_11_	406.380	3.106	451.000	179.000	[M-HCOO]^−^
2	loganin	C_17_H_26_O_10_	390.430	3.407	408.200	229.000	[M + NH_4_]^+^
3	baicalin	C_21_H_18_O_11_	446.360	4.592	445.100	269.000	[M + H]^−^
4	wogonoside	C_22_H_20_O_11_	460.390	5.100	461.100	283.000	[M + H]^+^
5	wogonin	C_16_H_12_O_5_	284.300	6.300	285.100	270.000	[M + H]^+^
6	sweroside	C_16_H_22_O_9_	358.340	3.490	357.100	125.000	[M-H]^−^

### Virtual docking and compound-target-pathway network analysis

**Fig. 3:**
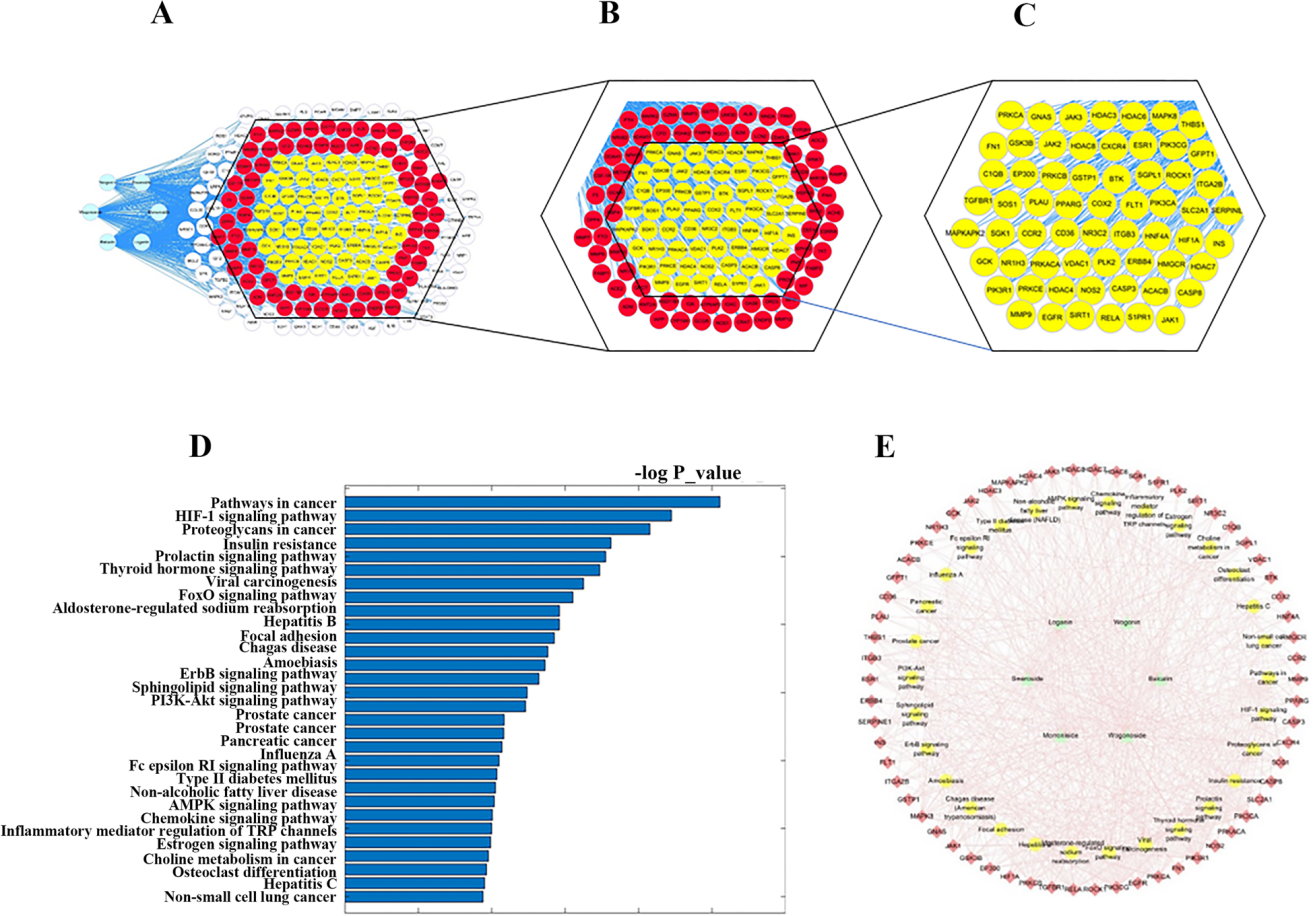
The results of network pharmacological analysis. **a** All results in the docking process. **b** Positive result in the docking process. **c** The targets selected with KEGG enrichment analysis. **d** Top 30 enrichment pathways of KEGG enrichment analysis. **e** Compound-target-pathway network. The node groups from inside to outside are molecule, pathway, and protein targets. The lines are interactions between each other

Molecular docking, KEGG enrichment analysis, and network analysis were applied. Molecular docking was a high-through method and a first filter tool for our study. In total, 373 targets were docked with 6 compounds after X-ray structures were filtered. After 2238 docking experiments, we acquired 748 positive results of molecule-target pairs, including 141 with morroniside, 141 with loganin, 163 with baicalin, 114 with wogonoside, 54 with wogonin, and 135 with sweroside. In virtual docking, molecules bound to targets via a series of interactive forces, such as hydrogen bonds, van der Waals forces, and conjugated bonds. The docking statistical results are shown in Fig. 3a–c. These targets were filtered in the form of molecular-target pairs, then 131 targets were used in KEGG enrichment analysis. After analysis and classification, 30 pathways entered the next step as potential active pathways. These enrichment pathways are shown in Fig. 3d.

By analyzing the representative pathways and targets under each term, a relationship of compound-target-pathway is displayed in a network shown in Fig. 3e. In this network, 97 nodes are marked, including 6 compounds, 61 target proteins, and 30 pathways. HNF4A, HMGCR, JAK3, and SIRT1 were outstanding according to the parameter “Neighborhood Connectivity”.

### Verification of the binding relationship of baicalin and wogonoside via SPR

**Fig. 4:**
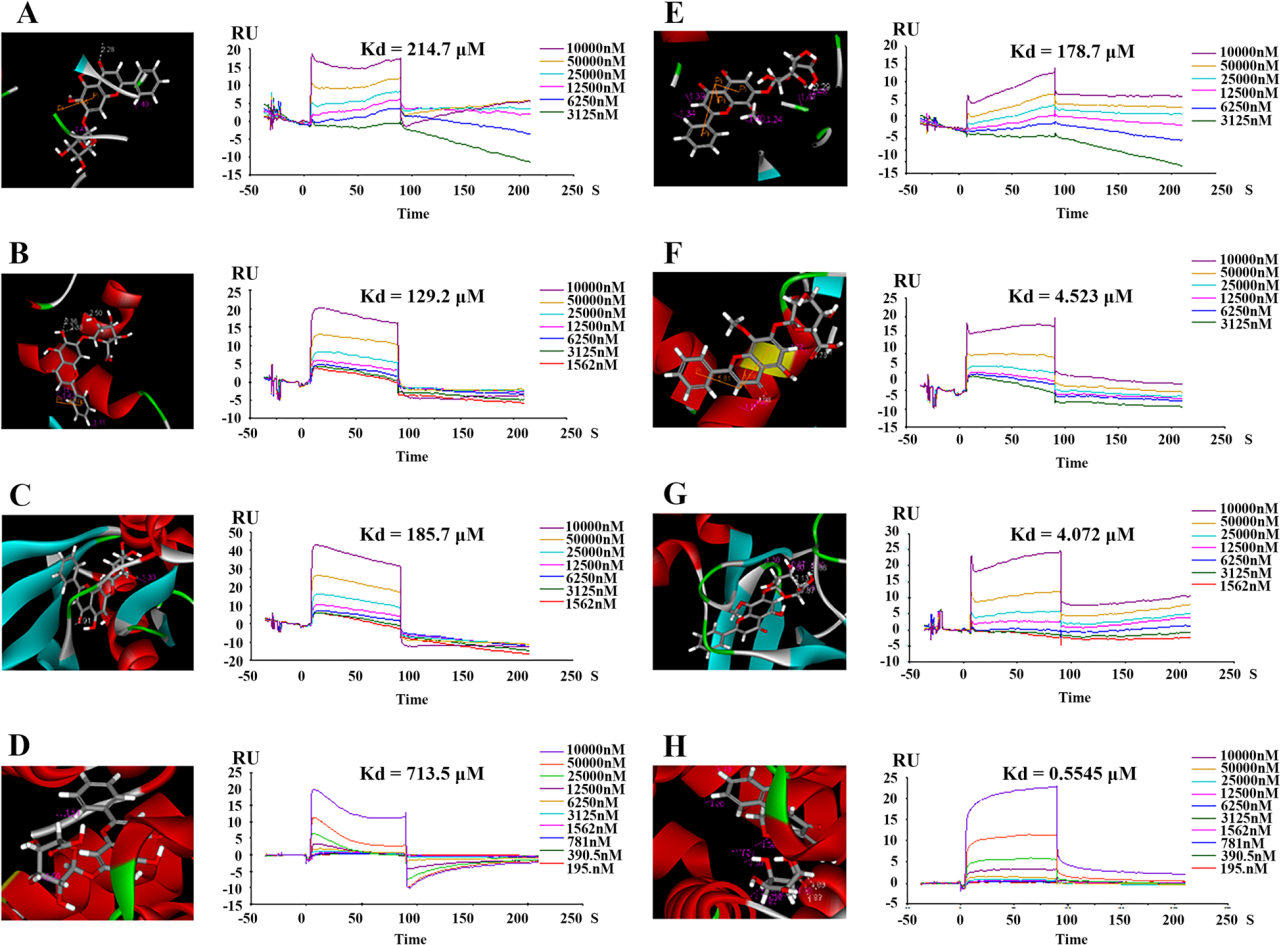
Binding diagram of eight representational molecule-target pairs. The left one in each group is the schematic drawing of virtual docking and the right is surface plasmon resonance (SPR) result. **a** NAD-dependent protein deacetylase sirtuin-1 (SIRT1) and baicalin. **b** 3-hydroxy-3-methylglutaryl-coenzyme A reductase (HMGCR) and baicalin. **c** Tyrosine-protein kinase Janus kinase 3 (JAK3) and baicalin. **d** Hepatocyte nuclear factor 4-alpha (HNF4A) and baicalin. **e** NAD-dependent protein deacetylase sirtuin-1 (SIRT1) and wogonoside. **f** 3-hydroxy-3-methylglutaryl-coenzyme A reductase (HMGCR) and wogonoside. **g** Tyrosine-protein kinase Janus kinase 3 (JAK3) and wogonoside. **h** Hepatocyte nuclear factor 4-alpha (HNF4A) and wogonoside

In total, 11 compound-target pairs were enrolled in the SPR test and they were shown in Table 3, including SIRT1 with morroniside, loganin, baicalin, wogonoside, and sweroside; HMGCR with baicalin and wogonoside; HNF4A with baicalin and wogonoside; and JAK3 with baicalin and wogonoside. Eight compound-target pairs, including baicalin and wogonoside with HNF4A, HMGCR, JAK3, and SIRT1, interacted with each other in vitro. All positive binding results are shown in Fig. 4a to h and the information and parameters of this result are shown in Table 4. Based on the result of SPR and KEGG enrichment analysis, we discovered that these four targets were clustered in the PI3K-AKT and AMPK signaling pathways. Baicalin and wogonoside were the most significantly compounds in our study.

**Table 3:** Notable targets after network analysis

Share Name	Common Name	PDB Code	Binding Molecule	Related pathway
HMGCR	3-hydroxy-3-methylglutaryl-coenzyme A reductase	1DQ8	baicalin; wogonoside	AMPK signaling pathway
HNF4A	Hepatocyte nuclear factor 4-alpha	3FS1	baicalin; wogonoside	AMPK signaling pathway
SIRT1	NAD-dependent protein deacetylase sirtuin-1	4ZZI	morroniside; loganin; baicalin; wogonoside; sweroside	AMPK signaling pathway
JAK3	Tyrosine-protein kinase Janus kinase 3	3LXL	baicalin; wogonoside	PI3K-Akt signaling pathway

**Table 4:** Positive result of surface plasmon resonance

Molecule	Protein	Libdockscore	Kd (μM)
baicalin	SIRT1	165.299	214.7
	HMGCR	144.197	129.2
	JAK3	146.95	185.7
	HNF4A	135.677	713.5
wogonoside	SIRT1	154.565	178.7
	HMGCR	144.063	4.523
	JAK3	134.219	4.072
	HNF4A	110.173	0.5545

### Baicalin and wogonoside enhanced cell viability in the HG condition in human podocyte cells

**Fig. 5:**
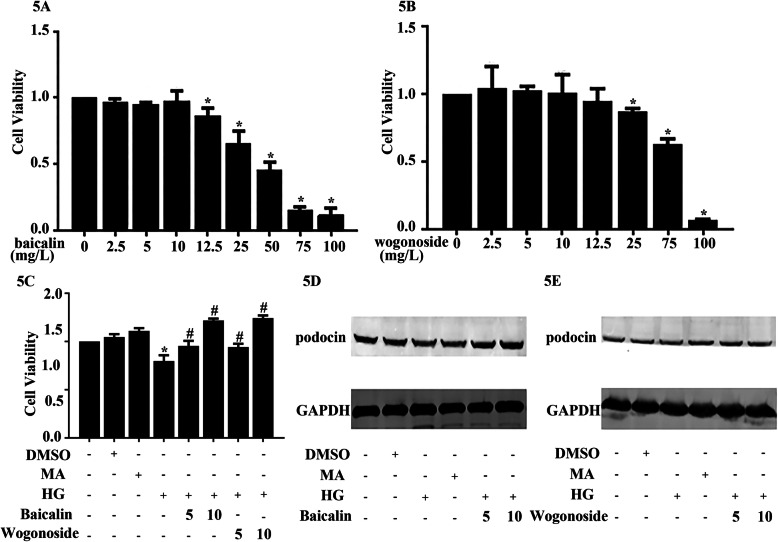
Baicalin and wogonoside enhanced cell viability in the high glucose condition in human podocyte cells. **a** Human podocyte cells were preconditioned with a series concentration of baicalin for 48 h and measured by Cell Count-8 assay. *Compared with 0 mg/L group, *n* = 3. **b** Human podocyte cells were preconditioned with a series concentration of wogonoside for 48 h and measured by Cell Count-8 assay. *Compared with 0 mg/L group, *n* = 3. **c** Human podocyte cells were treated with baicalin (5 or10 mg/L) or wogonoside (5 or10 mg/L) for 1 h and co-incubated with MO or HG for 48 h, then measured by Cell Count-8 assay. *Compared with control group. # Compared with HG group, *n* = 3. **d**, **e** Human podocyte cells were preconditioned with or without baicalin (5 or10 mg/L) or wogonoside (5 or 10 mg/L) for 1 h and treated with or without HG (30 mmol/L) for 24 h. The expression of podocin was tested by western blotting. Control: normal glucose condition (5 mM D-glucose); DMSO:dimethyl sulfoxide; HG: high glucose condition (30 mM D-glucose); MA (mannitol, 5 mM D-glucose + 25 mM mannitol)

To discover the influence of these compounds, we investigated the cytotoxicity of baicalin and wogonoside and discovered that they had little cytotoxicity to human podocytes at concentrations below 10 mg/L. This is shown in Fig. 5a and b. Furthermore, pretreating with baicalin and wogonoside at different doses offset the effect of HG on cell viability. The result suggested that HG induced the decreased cell viability; however, baicalin and wogonoside reduced this influence on human podocyte cells, as shown in Fig. 5c. We also verified this hypothesis using podocin, a marker of DN via western blotting, as shown in Fig. 5d, e.

### Baicalin and wogonoside altered the phosphorylation and expression of AMPK, PI3K, and AKT in the HG condition in human podocyte cells

**Fig. 6:**
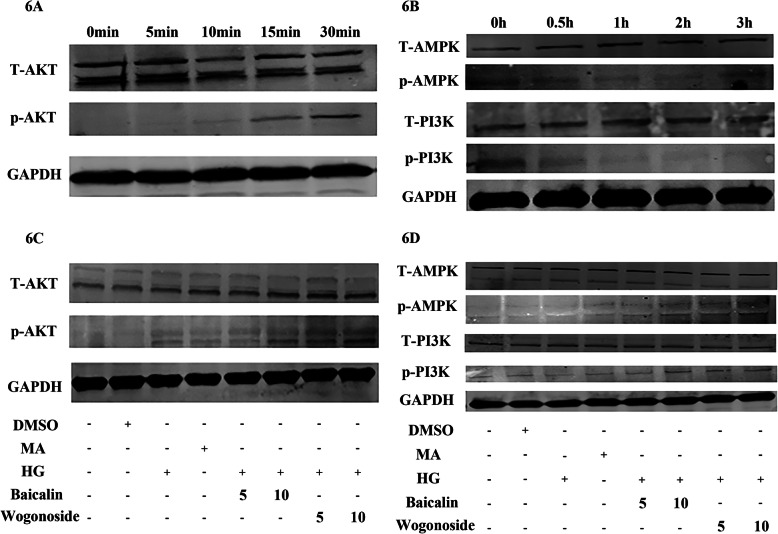
Baicalin and wogonoside altered the phosphorylation and expression of AMPK, PI3K, and AKT in HG-induced human podocyte cells. **a** Human podocyte cells were preconditioned with HG (30 mmol/L) for 0, 5, 10, 15, and 30 min and measured by western blotting. **b** Human podocyte cells were preconditioned with HG (30 mmol/L) for 0, 0.5, 1, 2, and 3 h, and measured by western blotting. **c** Human podocyte cells were preconditioned with baicalin (5 or10 mg/L) or wogonoside (5 or10 mg/L) for 12 h and incubated for 0.5 h with different treatments. The expression of AKT and p-AKT was measured by western blotting. **d** Human podocyte cells were preconditioned with baicalin (5 or10 mg/L) or wogonoside (5 or10 mg/L) for 12 h and incubated for 2 h with different treatments. The expression of AMPK, p-AMPK, PI3K, and p-PI3K was measured by western blotting. Control: normal glucose condition (5 mM D-glucose); DMSO:dimethyl sulfoxide; HG: high glucose condition (30 mM D-glucose); MA (mannitol, 5 mM D-glucose + 25 mM mannitol)

To verified influence of baicalin and wogonoside in PI3K-AKT pathway and AMPK pathway in cells, we used a western blot method. As a result, pretreatment with HG activated the phosphorylation of AKT and reduced the phosphorylation of AMPK and PI3K (Fig. 6a, b). However, baicalin and wogonoside blunted the HG-induced reduction of AMPK and PI3K phosphorylation. They also activated the expression of p-AKT directly, as shown in Fig. 6c, d.

## Discussion

DN, a diabetic complication, has become a worldwide challenge due to the lack of an effective treatment. Increased proteinuria is a typical clinical manifestation of DN and is mostly due to the damage of podocytes [20]. Podocytes are specialized epithelial cells that are present in the lateral glomerular basement membrane and retain the integrity of the glomerular filtration barrier [21]. Podocytes may be an important cell for DN research. In DN, unbalanced glucose metabolism causes abnormal expression of protein kinase C-alpha and mediates the reduction of nephrin in podocytes [22]. Moreover, HG may modulate matrix-related cell adhesion and integrin subunit synthesis, thereby affecting changes in podocyte and glomerular basement membrane interactions [23]. Under the pressure of HG, progressive podocyte injury continues and leads to the degeneration of podocyte foot processes, cell apoptosis, and loss of proteins [24].

The GDC may aid proteinuria reduction in combination with common treatments and has been used as a DN therapy for many years in China. However, to date, the pharmacological mechanism of GDC has not been defined due to the complex composition of compounds. Here, we used the methods of UHPLC-QQQ-MS/MS and network pharmacology to uncover the potential active compounds in GDC and preliminarily demonstrate the results in vitro. In our study, we built a molecular database including six compounds that could be absorbed into the blood of rata according to UHPLC-QQQ-MS/MS. A target database was also built. Molecular docking, KEGG enrichment analysis, and network analysis were performed and four notable targets, HNF4A, HMGCR, JAK3, and SIRT1, were observed. The binding relationship of molecules and these four targets were demonstrated via SPR. Finally, based on the result above, baicalin and wogonoside plobablely the active ingredients in GDC. And they might act via PI3K-AKT and AMPK signaling pathways. It also was observed that baicalin and wogonoside alleviated HG-induced decreased cell viability, protein marker podocin damage and adjust the protein expression of PI3K-AKT and AMPK signaling pathways in human podocyte cells.

Baicalin is a plant-derived flavonoid that has a potential antidiabetic effect [25]. Baicalin protects kidney epithelial cells against methylglyoxal-induced cytotoxicity via anti-inflammatory and antioxidant processes [26]. Baicalin can also reduce mitochondrial damage in streptozotocin-induced diabetic rats [27]. Meanwhile, wogonoside has anti-dyslipidemia, anti-obesity, and antidiabetic effects as a traditional Chinese medicine prescription [28]. Obviously, as we know, baicalin and wogonoside had protective action in DN progress.

And in SPR test, to our knowledge, four protein targets act on the progress of DN. HNF4A is a transcription factor that regulates glucose metabolism and nutrient-induced insulin secretion in pancreatic β-cells in mice [29]. It can also inhibit vascular endothelial growth factor (VEGF)-mediated endothelial proliferation and migration and reduce VEGF-stimulated in vitro angiogenesis by directly repressing the transcript of *FLK1* [30], which is closely correlated to the occurrence of DN. HMGCR is a therapeutic target and key enzyme in lipid metabolism [31]. Moreover, HMGCR inhibition, such as by atorvastatin [32], is one of the routine medicines for DN and can alleviate vascular oxidative stress [33]. JAK3 is an important molecular target for the treatment of autoimmune insulin-dependent (type 1) diabetes mellitus [34]. As a member of the JAK family, it also participates in the tyrosine-protein kinase Janus kinase /signal transducer and activator of transcription (JAK/STAT) pathway. The JAK/STAT pathway is involved in glucose metabolism [35] and T cell-reduced immune function [36]. SIRT1 has been shown to connect to β cell function in insulin secretion in vivo [37]. Moreover, some studies indicate that SIRT1 can alleviate diabetic albuminuria in DN and SIRT1 expression can be an early sign of DN [38]. In our study, the SPR test results indicated a correlation between baicalin and wogonoside with HNF4A, HMGCR, JAK3, and SIRT1 and DN, it might be the basis of baicalin and wogonoside’s protective action for cell damage in HG-induced podocyte cells.

Considering the pathways discovered to be involved in this research, PI3K-AKT and AMPK signaling pathways have a wide range of functions in the progress of DN. These pathways can be activated by advanced glycation end products, which play a central role in the disease pathophysiology of DN [39]. The activation of pathways can alter a series of physiological activities to counteract disease progress. For example, regarding the PI3K-AKT signaling pathway, a study demonstrated that PI3K-AKT mediates the inhibitory effects of oxidative stress and inflammation in chronic kidney disease [40]. Another study suggested that the PI3K-AKT pathway is involved in HG-induced autophagy and apoptosis in human proximal tubular epithelial cells [41]. The targets in the AMPK signaling pathway, especially AMPK, are also involved in the protection of mitochondrial function in diabetic microvascular complications [42], such as DN. Additionally, AMPK participates in podocyte cell apoptosis as an upstream protein that regulates the mammalian target of rapamycin complex 1 [43]. As a preliminary study, our research showed the adjustment of baicalin and wogonoside in PI3K-AKT and AMPK signaling pathways. However, it still need a further study to find how drugs act through these two pathways.

In conclusion, baicalin and wogonoside may protect HG-induced podocytes against damage by influencing the AMPK and PI3K-AKT signaling pathways via binding with HNF4A, HMGCR, JAK3, and SIRT1. This study can be used to elucidate the mechanism of action of GDC for DN in future research.

## Conclusion

In this study, six compounds in GDC were identified using a standard comparison method via UHPLC-QQQ-MS/MS. After docking, enrichment analysis and network analysis, 11 compound-target pairs were selected to SPR. The results suggested that baicalin and wogonoside bound with HNF4A, HMGCR, JAK3, and SIRT1. Moreover, baicalin and wogonoside prevented HG-induced decreased cell viability and podocin reduction in podocytes. They also altered the phosphorylation of AMPK, PI3K, and AKT. Therefore, the influence of baicalin and wogonoside in PI3K-AKT and AMPK signaling pathways by binding with HNF4A, HMGCR, JAK3, and SIRT1 may be one of the aspects involved in the mechanism of action of GDC for the treatment of DN. This study can be used to inform future mechanistic research into GDC, DN, and other TCM.

## Supplementary Information

**Additional file 1:** **Table S1.** The information of eight traditional Chinese herbs of GDC.

**Additional file 2:** **Table S2.** 97 components in molecule database.

**Additional file 3. **Network analysis data.

**Additional file 4. **Western Blot.

## Data Availability

The datasets used and/or analyzed during the current study are available from the corresponding author on reasonable request.

## Abbreviations

DN: Diabetic nephrophy
SGLT-2: Sodium-dependent glucose transporter 2
ACEI: Angiotensin-converting enzyme inhibitor
GDC: Gandi capsule
TCM: Traditional Chinese medicine
eGFR: Estimated glomerular filtration rate
SPR: Surface plasmon resonance
SD: Sprague Dawley
PI3K: Phosphoinositide 3-kinase
AKT: Protein kinase B
AMPK: 5′ AMP-activated protein kinase
HNF4A: Hepatocyte nuclear factor 4-alpha
HMGCR: 3-hydroxy-3-methylglutaryl-coenzyme A reductase
JAK3: Tyrosine-protein kinase Janus kinase 3
SIRT1: NAD-dependent protein deacetylase sirtuin-1
CUR: Curtain
CAD: Collision activation dissociation
MRM: Multiple reaction monitoring
DP: Declustering potential
EP: Entrance potential
CE: Collision energy
CXP: Collision cell exit potentials
OB: Oral bioavailability
DL: Drug-likeness
TTD: Therapeutic Target Database
GEO: Gene Expression Omnibus
logFC: Log fold change
KEGG: Kyoto Encyclopedia of Genes and Genomes
NC: Neighborhood Connectivity
RPMI: Roswell Park Memorial Institute
FBS: Fetal bovine serum
CCK-8: Cell counting kit-8
HG: High glucose
MA: Mannitol
DMSO: Dimethyl sulfoxide
TBST: 1× Tris-buffered saline and 0.1% Tween 20
VEGF: Vascular endothelial growth factor
STAT: Signal transducer and activator of transcription
