# Supplementary material for: Revealing active components, action targets and molecular mechanism of Gandi capsule for treating diabetic nephropathy based on network pharmacology strategy
Source: BMC Complement Med Ther. 2020 Nov 23;20:362. doi: 10.1186/s12906-020-03155-4 (PMC7685593; doi:10.1186/s12906-020-03155-4)
Supplement: Supplementary file 1 — Additional file 1: Table S1. The information of eight traditional Chinese herbs of GDC. [file 12906_2020_3155_MOESM1_ESM.docx]

Table S1. The information of eight traditional Chinese herbs of GDC

|  | Latin name | Plant sources | Plant part used |
| --- | --- | --- | --- |
| 1 | Astragali Radix | Astragalus mongholicus Bunge. | root |
| 2 | Corni Fructus | Cornus officinalis Siebold & Zucc | fruit |
| 3 | Rehmanniae Radix | Rehmannia glutinosa Gaertn. | root |
| 4 | Leonuri Herba | Leonurus japonicus Houtt. | whole grass |
| 5 | Sophorae Flos | Styphnolobium japonicum (L.) Schott. | flower |
| 6 | Scutellariae Radix | Scutellaria baicalensis Georgi. | root |
| 7 | Bombyx Batryticatus | Bombyx scindicus (Stocks) I.Riedl. | whole worm |
| 8 | phyllanthi fructus | Phyllanthus emblica L. | fruit |
