# Supplementary material for: Revealing active components, action targets and molecular mechanism of Gandi capsule for treating diabetic nephropathy based on network pharmacology strategy
Source: BMC Complement Med Ther. 2020 Nov 23;20:362. doi: 10.1186/s12906-020-03155-4 (PMC7685593; doi:10.1186/s12906-020-03155-4)
Supplement: Supplementary file 2 — Additional file 2: Table S2. 97 components in molecule database. [file 12906_2020_3155_MOESM2_ESM.docx]

**Table S2.** 97 components in molecule database

| Mol ID | Molecule Name | MW | AlogP | OB (%) | Caco-2 | BBB | DL | HL | Source |
| --- | --- | --- | --- | --- | --- | --- | --- | --- | --- |
| MOL000449 | Stigmasterol | 412.77 | 7.64 | 43.83 | 1.44 | 1 | 0.76 | 5.57 | dihuang |
| MOL000359 | sitosterol | 414.79 | 8.08 | 36.91 | 1.32 | 0.87 | 0.75 | 5.37 | dihuang |
| MOL000358 | beta-sitosterol | 414.79 | 8.08 | 36.91 | 1.32 | 0.99 | 0.75 | 5.36 | huaihua |
| MOL000354 | isorhamnetin | 316.28 | 1.76 | 49.6 | 0.31 | -0.54 | 0.31 | 14.34 | huaihua |
| MOL000422 | kaempferol | 286.25 | 1.77 | 41.88 | 0.26 | -0.55 | 0.24 | 14.74 | huaihua |
| MOL000033 | (3S,8S,9S,10R,13R,14S,17R)-10,13-dimethyl-17-[(2R,5S)-5-propan-2-yloctan-2-yl]-2,3,4,7,8,9,11,12,14,15,16,17-dodecahydro-1H-cyclopenta[a]phenanthren-3-ol | 428.82 | 8.54 | 36.23 | 1.45 | 1.09 | 0.78 | 5.22 | huangqi |
| MOL000296 | hederagenin | 414.79 | 8.08 | 36.91 | 1.32 | 0.96 | 0.75 | 5.35 | huangqi |
| MOL000371 | 3,9-di-O-methylnissolin | 314.36 | 2.89 | 53.74 | 1.18 | 0.63 | 0.48 | 9 | huangqi |
| MOL000378 | 7-O-methylisomucronulatol | 316.38 | 3.38 | 74.69 | 1.08 | 0.84 | 0.3 | 2.98 | huangqi |
| MOL000438 | (3R)-3-(2-hydroxy-3,4-dimethoxyphenyl)chroman-7-ol | 302.35 | 3.13 | 67.67 | 0.96 | 0.34 | 0.26 | 2.9 | huangqi |
| MOL000380 | (6aR,11aR)-9,10-dimethoxy-6a,11a-dihydro-6H-benzofurano[3,2-c]chromen-3-ol | 300.33 | 2.64 | 64.26 | 0.93 | 0.55 | 0.42 | 8.49 | huangqi |
| MOL000442 | 1,7-Dihydroxy-3,9-dimethoxy pterocarpene | 314.31 | 3.11 | 39.05 | 0.89 | -0.04 | 0.48 | 7.95 | huangqi |
| MOL000392 | formononetin | 268.28 | 2.58 | 69.67 | 0.78 | 0.02 | 0.21 | 17.04 | huangqi |
| MOL000211 | Mairin | 456.78 | 6.52 | 55.38 | 0.73 | 0.22 | 0.78 | 8.87 | huangqi |
| MOL000239 | Jaranol | 314.31 | 2.09 | 50.83 | 0.61 | -0.22 | 0.29 | 15.5 | huangqi |
| MOL000398 | isoflavanone | 316.33 | 2.42 | 109.99 | 0.53 | 0.17 | 0.3 | 15.51 | huangqi |
| MOL000417 | Calycosin | 284.28 | 2.32 | 47.75 | 0.52 | -0.43 | 0.24 | 17.1 | huangqi |
| MOL000387 | Bifendate | 418.38 | 2.56 | 31.1 | 0.15 | -0.06 | 0.67 | 17.96 | huangqi |
| MOL000098 | quercetin | 302.25 | 1.5 | 46.43 | 0.05 | -0.77 | 0.28 | 14.4 | huangqi |
| MOL000379 | 9,10-dimethoxypterocarpan-3-O-β-D-glucoside | 462.49 | 0.74 | 36.74 | -0.63 | -1.5 | 0.92 | 13.06 | huangqi |
| MOL002776 | Baicalin | 446.39 | 0.64 | 40.12 | -0.85 | -1.74 | 0.75 | 17.36 | huangqi |
| MOL000433 | FA | 441.45 | 0.01 | 68.96 | -1.5 | -2.59 | 0.71 | 24.81 | huangqi |
| MOL000439 | isomucronulatol-7,2'-di-O-glucosiole | 626.67 | -0.68 | 49.28 | -2.22 | -3.36 | 0.62 | 0.93 | huangqi |
| MOL000374 | 5'-hydroxyiso-muronulatol-2',5'-di-O-glucoside | 642.67 | -0.95 | 41.72 | -2.47 | -3.62 | 0.69 | 2.52 | huangqi |
| MOL005486 | 3,4-Dehydrolycopen-16-al | 548.92 | 11.48 | 46.64 | 2 | 0.6 | 0.49 | 4.29 | shanzhuyu |
| MOL005481 | 2,6,10,14,18-pentamethylicosa-2,6,10,14,18-pentaene | 342.67 | 9.51 | 33.4 | 1.94 | 1.99 | 0.24 | 6.05 | shanzhuyu |
| MOL001495 | Ethyl linolenate | 306.54 | 6.55 | 46.1 | 1.54 | 1.12 | 0.2 | 6.2 | shanzhuyu |
| MOL001494 | Mandenol | 308.56 | 6.99 | 42 | 1.46 | 1.14 | 0.19 | 5.39 | shanzhuyu |
| MOL005557 | lanosta-8,24-dien-3-ol,3-acetate | 468.84 | 8.5 | 44.3 | 1.45 | 1.31 | 0.82 | 7.21 | shanzhuyu |
| MOL001771 | poriferast-5-en-3beta-ol | 414.79 | 8.08 | 36.91 | 1.45 | 1.14 | 0.75 | 5.07 | shanzhuyu |
| MOL002883 | Ethyl oleate (NF) | 310.58 | 7.44 | 32.4 | 1.4 | 1.1 | 0.19 | 4.85 | shanzhuyu |
| MOL008457 | Tetrahydroalstonine | 352.47 | 2.66 | 32.42 | 0.9 | 0.33 | 0.81 | 10.55 | shanzhuyu |
| MOL002879 | Diop | 390.62 | 7.44 | 43.59 | 0.79 | 0.26 | 0.39 | 3.6 | shanzhuyu |
| MOL005530 | Hydroxygenkwanin | 300.28 | 2.32 | 36.47 | 0.52 | -0.44 | 0.27 | 15.22 | shanzhuyu |
| MOL001680 | Loganin | 390.43 | -2.08 | 35.9 | -1.48 | -2.26 | 0.44 | 0 | shanzhuyu |
| MOL005503 | Cornudentanone | 378.56 | 4.97 | 39.66 | 0.47 | 0.09 | 0.33 | 2.83 | shanzhuyu |
| MOL000650 | Sweroside | 358.38 | -1.59 | 34.96 | -1.08 | -1.43 | 0.38 | 0 | shanzhuyu |
| MOL005360 | malkangunin | 432.56 | 1.84 | 57.71 | 0.22 | -0.17 | 0.63 | 4.09 | shanzhuyu |
| MOL005531 | Telocinobufagin | 402.58 | 2.11 | 69.99 | -0.12 | -0.85 | 0.79 | 5.15 | shanzhuyu |
| MOL003137 | Leucanthoside | 462.44 | -0.07 | 32.12 | -1.27 | -2.41 | 0.78 | 16.28 | shanzhuyu |
| MOL005489 | 3,6-Digalloylglucose | 484.4 | -0.03 | 31.42 | -1.95 | -3.05 | 0.66 | 3.39 | shanzhuyu |
| MOL000554 | gallic acid-3-O-(6'-O-galloyl)-glucoside | 484.4 | -0.03 | 30.25 | -1.96 | -2.76 | 0.67 | 2.48 | shanzhuyu |
| MOL005552 | gemin D | 634.49 | 0.73 | 68.83 | -2.17 | -2.71 | 0.56 | 5.55 | shanzhuyu |
| MOL001683 | morroniside | 244.27 | -0.72 | 31.68 | -0.6 | -1.25 | 0.2 | 0 | shanzhuyu |
| MOL001420 | ZINC04073977 | 412.77 | 7.76 | 38 | 1.46 | 1.27 | 0.76 | 5.45 | yimucao |
| MOL001439 | arachidonic acid | 304.52 | 6.41 | 45.57 | 1.2 | 0.58 | 0.2 | 4.39 | yimucao |
| MOL001421 | preleoheterin | 334.5 | 1.87 | 85.97 | 0.46 | 0.29 | 0.33 | 2.98 | yimucao |
| MOL001422 | iso-preleoheterin | 334.5 | 2 | 66.29 | 0.44 | 0.36 | 0.33 | 3.08 | yimucao |
| MOL001418 | galeopsin | 376.54 | 3.28 | 61.02 | 0.42 | -0.04 | 0.38 | -10.26 | yimucao |
| MOL006824 | α-amyrin | 426.8 | 7.35 | 39.51 | 1.37 | 1.2 | 0.76 | 3.06 | yuganzi |
| MOL006812 | Phyllanthin | 418.58 | 4.11 | 33.31 | 1.06 | 0.57 | 0.42 | 3.36 | yuganzi |
| MOL000006 | luteolin | 286.25 | 2.07 | 36.16 | 0.19 | -0.84 | 0.25 | 15.94 | yuganzi |
| MOL000492 | (+)-catechin | 290.29 | 1.92 | 54.83 | -0.03 | -0.73 | 0.24 | 0.61 | yuganzi |
| MOL001002 | ellagic acid | 302.2 | 1.48 | 43.06 | -0.44 | -1.41 | 0.43 | -1.04 | yuganzi |
| MOL005983 | leucodelphinidin | 322.29 | 0.82 | 43.45 | -0.48 | -1.23 | 0.31 | 0.6 | yuganzi |
| MOL006821 | (-)-epigallocatechin-3-gallate | 458.4 | 2.89 | 55.09 | -0.57 | -1.7 | 0.77 | 1.7 | yuganzi |
| MOL000569 | digallate | 322.24 | 1.53 | 61.85 | -0.76 | -1.52 | 0.26 | 5.29 | yuganzi |
| MOL006801 | phyllaemblicacid methyl ester | 436.45 | 0.93 | 43.09 | -0.86 | -1.13 | 0.73 | 9.76 | yuganzi |
| MOL006826 | chebulic acid | 356.26 | -0.26 | 72 | -1.4 | -1.75 | 0.32 | 3.44 | yuganzi |
| MOL006796 | mucic acid 1,4-lactone 5-0-gallate | 344.25 | -0.67 | 52.26 | -1.73 | -2.29 | 0.27 | 7.01 | yuganzi |
| MOL006793 | mucic acid 1,4-lactone 2-0-gallate | 358.28 | -0.19 | 49.56 | -1.86 | -2.22 | 0.31 | 4.68 | yuganzi |
| MOL006799 | (2S,3R,3aS,4R,4'S,5'R,6S,7aR)-3,4,4'-trihydroxy-3,5'-bis(hydroxymethyl)spiro[3a,4,5,6,7,7a-hexahydrobenzofuran-2,2'-tetrahydropyran]-6-carboxylic acid | 348.39 | -2.55 | 48.46 | -1.92 | -2.64 | 0.31 | 4.98 | yuganzi |
| MOL006806 | Phyllanemblinin A | 616.47 | 1.42 | 72.44 | -2.28 | -3.14 | 0.33 | 19.43 | yuganzi |
| MOL006802 | phyllaemblicin A | 582.61 | -1.19 | 45.63 | -2.37 | -2.62 | 0.77 | 11.56 | yuganzi |
| MOL013068 | Wogonoside | 459.41 | 0.21 | 37.07 | -1.68 | -2.15 | 0.77 | 0 | huangqin |
| MOL000173 | wogonin | 284.28 | 2.59 | 30.68 | 0.79 | 0.04 | 0.23 | 17.75 | huangqin |
| MOL001689 | acacetin | 284.28 | 2.59 | 34.97 | 0.67 | -0.05 | 0.24 | 17.25 | huangqin |
| MOL000228 | (2R)-7-hydroxy-5-methoxy-2-phenylchroman-4-one | 270.3 | 2.82 | 55.23 | 0.87 | 0.26 | 0.2 | 17.02 | huangqin |
| MOL002714 | baicalein | 270.25 | 2.33 | 33.52 | 0.63 | -0.05 | 0.21 | 16.25 | huangqin |
| MOL002908 | 5,8,2'-Trihydroxy-7-methoxyflavone | 300.28 | 2.32 | 37.01 | 0.76 | -0.07 | 0.27 | 16.17 | huangqin |
| MOL002909 | 5,7,2,5-tetrahydroxy-8,6-dimethoxyflavone | 376.34 | 2.02 | 33.82 | 0.35 | -0.59 | 0.45 | 15.94 | huangqin |
| MOL002910 | Carthamidin | 288.27 | 2.03 | 41.15 | 0.16 | -0.42 | 0.24 | 15.81 | huangqin |
| MOL002911 | 2,6,2',4'-tetrahydroxy-6'-methoxychaleone | 302.3 | 2.62 | 69.04 | -0.07 | -0.32 | 0.22 | 21.89 | huangqin |
| MOL002913 | Dihydrobaicalin_qt | 272.27 | 2.3 | 40.04 | 0.56 | 0.18 | 0.21 | 16.13 | huangqin |
| MOL002914 | Eriodyctiol (flavanone) | 288.27 | 2.03 | 41.35 | 0.05 | -0.66 | 0.24 | 15.88 | huangqin |
| MOL002915 | Salvigenin | 328.34 | 2.82 | 49.07 | 0.86 | -0.03 | 0.33 | 15.87 | huangqin |
| MOL002917 | 5,2',6'-Trihydroxy-7,8-dimethoxyflavone | 330.31 | 2.3 | 45.05 | 0.48 | -0.11 | 0.33 | 16.37 | huangqin |
| MOL002925 | 5,7,2',6'-Tetrahydroxyflavone | 286.25 | 2.07 | 37.01 | 0.18 | -0.56 | 0.24 | 18 | huangqin |
| MOL002926 | dihydrooroxylin A | 286.3 | 2.55 | 38.72 | 0.71 | 0.03 | 0.23 | 17.58 | huangqin |
| MOL002927 | Skullcapflavone II | 374.37 | 2.54 | 69.51 | 0.68 | -0.07 | 0.44 | 16.14 | huangqin |
| MOL002928 | oroxylin a | 284.28 | 2.59 | 41.37 | 0.76 | 0.13 | 0.23 | 17.15 | huangqin |
| MOL002932 | Panicolin | 314.31 | 2.57 | 76.26 | 0.84 | 0.31 | 0.29 | 16.78 | huangqin |
| MOL002933 | 5,7,4'-Trihydroxy-8-methoxyflavone | 300.28 | 2.32 | 36.56 | 0.46 | -0.4 | 0.27 | 16.93 | huangqin |
| MOL002934 | NEOBAICALEIN | 374.37 | 2.54 | 104.34 | 0.74 | -0.19 | 0.44 | 16.5 | huangqin |
| MOL002937 | DIHYDROOROXYLIN | 286.3 | 2.55 | 66.06 | 0.67 | 0.13 | 0.23 | 17.17 | huangqin |
| MOL000525 | Norwogonin | 270.25 | 2.33 | 39.4 | 0.6 | -0.17 | 0.21 | 16.93 | huangqin |
| MOL000552 | 5,2'-Dihydroxy-6,7,8-trimethoxyflavone | 344.34 | 2.55 | 31.71 | 0.93 | 0 | 0.35 | 16.47 | huangqin |
| MOL000073 | ent-Epicatechin | 290.29 | 1.92 | 48.96 | 0.02 | -0.64 | 0.24 | 0.63 | huangqin |
| MOL001458 | coptisine | 320.34 | 3.25 | 30.67 | 1.21 | 0.32 | 0.86 | 9.33 | huangqin |
| MOL001490 | bis[(2S)-2-ethylhexyl] benzene-1,2-dicarboxylate | 390.62 | 7.57 | 43.59 | 0.98 | 0.68 | 0.35 | 3.02 | huangqin |
| MOL001506 | Supraene | 410.8 | 11.33 | 33.55 | 2.08 | 1.73 | 0.42 | 2.72 | huangqin |
| MOL002897 | epiberberine | 336.39 | 3.45 | 43.09 | 1.17 | 0.4 | 0.78 | 6.1 | huangqin |
| MOL008206 | Moslosooflavone | 298.31 | 2.84 | 44.09 | 1.01 | 0.54 | 0.25 | 17.02 | huangqin |
| MOL010415 | 11,13-Eicosadienoic acid, methyl ester | 322.59 | 7.55 | 39.28 | 1.46 | 1.24 | 0.23 | 5.44 | huangqin |
| MOL012245 | 5,7,4'-trihydroxy-6-methoxyflavanone | 302.3 | 2.28 | 36.63 | 0.43 | -0.32 | 0.27 | 16.12 | huangqin |
| MOL012246 | 5,7,4'-trihydroxy-8-methoxyflavanone | 302.3 | 2.28 | 74.24 | 0.37 | -0.43 | 0.26 | 16.85 | huangqin |
| MOL012266 | rivularin | 344.34 | 2.55 | 37.94 | 0.65 | -0.13 | 0.37 | 16.25 | huangqin |
